# Exploring the Impact of Cytogenetic Abnormalities on Treatment Responses and Survival Outcomes in Multiple Myeloma: A Single-Centre Experience of 13 Years of Follow-Up

**DOI:** 10.3390/biomedicines12051014

**Published:** 2024-05-04

**Authors:** Mehmet Ali Kazgı, Ertugrul Bayram, Tolga Kosecı, Burak Mete, Tugba Toyran, Melek Ergin, Ismail Oguz Kara

**Affiliations:** 1 Department of Internal Medicine, Faculty of Medicine, Cukurova University, Adana 01250, Turkey; mehmetali.kazgi.mak81@gmail.com; 2 Department of Medical Oncology, Faculty of Medicine, Cukurova University, Adana 01250, Turkey; drtolgakoseci@gmail.com (T.K.); iokara@cu.edu.tr (I.O.K.); 3 Department of Public Health, Faculty of Medicine, Cukurova University, Adana 01250, Turkey; burakmete2008@gmail.com; 4 Department of Medical Pathology, Faculty of Medicine, Cukurova University, Adana 01250, Turkey; tugbaolcan@hotmail.com (T.T.); erginm@cu.edu.tr (M.E.)

**Keywords:** multiple myeloma, cytogenetic abnormalities, immunomodulators

## Abstract

(1) Background: The introduction of novel therapies has led to a considerable evolution in the management of Multiple Myeloma, and chromosomal abnormalities predict the success of treatment. We aimed to characterize cytogenetic abnormalities for risk stratification in the patient population and to evaluate the predictive and prognostic value of the specified abnormalities in distinct treatment modalities. (2) Methods: This study included patients with Multiple Myeloma who applied to the Internal Medicine Clinic of the Cukurova University Faculty of Medicine. Between 2010 and 2023, 98 cases with cytogenetic abnormality data were identified. We analysed the effects of cytogenetic abnormalities on survival and response rates to first chemotherapies. (3) Results: P53 del was the most prevalent abnormality, and t(11;14) was the most common translocation. There was no significant difference in the mean survival and treatment response rates for specific cytogenetic abnormalities. When chemotherapies based on lenalidomide were initiated, patients’ life-death statuses differed significantly from those of treatments without lenalidomide. Regardless of the type of chromosomal aberration, lenalidomide-based treatments independently enhanced average survival 14-fold, while there was no significant difference in overall survival among treatments. (4) Conclusions: In individuals with cytogenetic abnormalities, lenalidomide-based treatments should be started regardless of the chemotherapy to be used for the condition.

## 1. Introduction

Multiple Myeloma (MM) is a plasma cell dyscrasia characterised by the excessive proliferation of plasma cells in the bone marrow and hyperviscosity due to paraproteinemia. MM accounts for 1% of all cancers and approximately 10% of haematological cancers [1,2].

In MM, the average lifespan is nearly ten years [3]. Cytogenetic abnormalities play an important role in the prognosis of the disease and are assessed at the time of diagnosis [4]. The most common cytogenetic abnormality is trisomy. Common chromosomal abnormalities in individuals with MM are abnormalities on chromosome 13, which have been reported to account for 10–12% of all chromosome losses and to be seen in 40–50% of cases when evaluated with standard cytogenetic methods and fluorescence in situ hybridization (FISH) [5]. The most common translocation is t(11;14), followed by t(4;14). Based on the risk group, cytogenetic factors are categorized as either high or low risk. Trisomies, t(11;14), and t(6;14) are in the low-risk group, while t(4;14), t(14;16), t(14;20), amp(1q), and del(17p) are in the high-risk group, according to the Mayo Clinic’s risk categorization [6]. Clinical research indicates that MM patients with high-risk cytogenetic markers have an average life expectancy of only five years compared to over ten years for the low-risk group [6].

Treatment efficacy has been markedly improved by the introduction of new molecules into the MM therapeutic landscape over the past 15 years, as well as by their synergistic use with traditional treatments. The introduction of dicitosteroids like dexamethasone and prednisolone represents the continued integration of well-established therapy alternatives from the past into the modern therapeutic arsenal, even though alkylating drugs like melphalan and cyclophosphamide are still used [7,8]. With the introduction of proteasome and immunomodulatory drugs in MM treatment, 5-year survival rates have increased. Today, clinics specialised in MM report median survival rates of over 7 years. However, despite many favourable findings in treatment and follow-up, MM is still not a curable disease, and patients are evaluated to determine their eligibility for autologous stem cell transplantation [9].

MM tumour characteristics, which represent the disease’s natural progression and treatment sensitivity, are determined by genetic variations of MM [10]. This study represents a survey of patients followed up with by our clinic over the last 13 years. During this time, we aimed to identify cytogenetic abnormalities involved in the risk stratification of patients screened using FISH (fluorescence in situ hybridization) and NGS (next-generation sequencing) techniques and in whom cytogenetic abnormalities were detected. The focus of our research is to evaluate the predictive potential and prognostic significance of these identified abnormalities in specific treatment modalities.

## 2. Materials and Methods

### 2.1. Study Design and Participants

**Figure 1:**
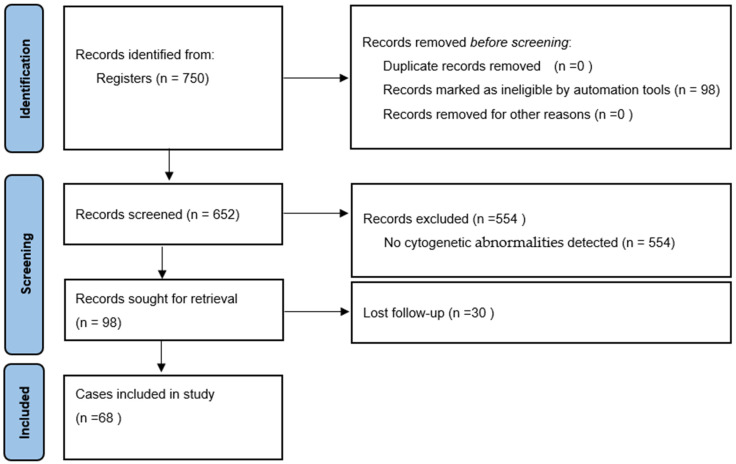
Flow chart for selection of patients.

In this retrospective cohort study, we identified 652 patients with Multiple Myeloma diagnosed at the Cukurova University Faculty of Medicine Internal Medicine Outpatient Clinic between January 2010 and January 2023. Patients who met the criteria were identified from the database of our hospital, the Medical Pathology Department, the Medical Genetics Department, and archived files (Figure 1).

### 2.2. Inclusion and Exclusion Criteria

Patients included in the study met the following criteria: (1) a diagnosis of multiple myeloma (MM) with cytogenetic abnormalities; (2) the availability of their complete medical records, including KT, laboratory, and imaging data; (3) the receipt of chemotherapy at Çukurova University Faculty of Medicine Balcalı Hospital; and (4) receiving ongoing follow-up care at our institution. Patients were excluded if they (1) lacked the necessary data for analysis; (2) had previously received chemotherapy for MM; or (3) their medical records were incomplete or inaccessible. Additionally, individuals with (4) plasma cell malignancies other than MM and those who were (5) diagnosed with monoclonal gammopathy of undetermined significance (MGUS) and smouldering multiple myeloma (SMM), were not included in the study.

Treatment regimens involved a combination of chemotherapy and immunomodulatory drugs and represent standard protocols used in the treatment of Multiple Myeloma, which are VD (a combination therapy consisting of bortezomib and dexamethasone), VCD (a combination therapy consisting of bortezomib, cyclophosphamide, and dexamethasone), VRD (a combination therapy consisting of bortezomib, lenalidomide, and dexamethasone), or VAD (a combination therapy consisting of bortezomib, doxorubicin, and dexamethasone)

### 2.3. Risk Scale (R-ISS)

The R-ISS stage was determined according to LDH, albumin, and β2 microglobulin values and cytogenetic abnormalities at the time of diagnosis [11]. Cytogenetic abnormalities detected at the time of diagnosis or in subsequent investigations were included in the initial diagnosis [12]. The first chemotherapies received after the diagnosis and response rates to these chemotherapies were analysed. Progression status, survival, the effect of cytogenetic abnormalities on survival, and response rates to chemotherapy were collected and recorded in our database.

### 2.4. Cytogenetic Abnormalities Analyses

We identified patients with cytogenetic abnormalities screened by FISH and NGS. Data were obtained from the Departments of Medical Pathology and Medical Genetics. A total of 98 cases with cytogenetic abnormalities were identified, and only 68 cases were eligible for evaluation. Laboratory values (LDH, albumin, β2 microglobulin, free Kappa–Lambda, Cre, Sedim, total protein, and immunglobulin) were obtained from these data.

Samples of BM or peripheral blood were studied following the FISH procedures (Abbott Molecular, Abbott Park, IL, USA). Glass slides were affixed with the target DNA. For five minutes at 73 °C, the DNA and probe-containing slide were denatured in 70% formamide (pH 7.0). After running the slides through a batch of cold ethanol, the slides were left to dry. After applying the denatured probe to the target DNA, the slides were left to incubate for an entire night at 37 °C. Following hybridization, they were rinsed twice for three minutes at room temperature in phosphate-buffered detergent; then, the slides were dyed for two minutes. After that, a fluorescence microscope (Bx52, Olympus, Tokyo, Japan) fitted with suitable filters was used to view the slides.

A capture panel was used for NGS in order to sequence data on the Illumina NextSeq platform. Following plasma cell enrichment, samples containing at least 80% plasma cells in the final cell pellet were used to extract DNA. DNA extracted from samples was kept in RLT+ buffer (Qiagen) using the Qiagen DNA/RNA kit (Venlo, The Netherlands).

### 2.5. Treatment Response Assessment

The response status of the patients to chemotherapy was considered treatment-responsive if their BM plasma cell rate was <5% and the patients were found to respond via laboratory and imaging methods. Patients with a control BM plasma cell ratio of >5% and those who were found to be non-responsive as a result of laboratory and imaging methods, those whose treatment process continued, and deceased patients were considered treatment non-responsive/refractory.

### 2.6. Statistical Analysis

The conformity of continuous variables to a normal distribution was analysed by visual (histogram) and analytical methods (Kolmogorov–Smirnov). In descriptive analyses, mean and standard deviations were used for normally distributed variables, while median and interquartile range values were given for non-normally-distributed variables. An independent-samples t-test was used for the comparison of normally distributed numerical variables. Chi-Squared or Fisher’s exact tests were used to evaluate the relationships between parameters according to bone marrow response status and survival–deceased status. The relationship between patient clinical characteristics and survival times was evaluated using Kaplan–Meier survival analysis and log-rank test. SPSS 20.0 (IBM Corp., Armonk, NY, USA) software was used to evaluate the analyses, and *p* < 0.05 was accepted for statistical significance.

## 3. Results

### Patient Characteristics

It was observed that 36.8% of the patients (25 patients) were female and 63.2% (43 patients) were male. It was observed that 32% of the patients were <65 years old; 67.6% were >65 years old. The median age of our patients was 59.0 years. It was determined that 80.9% of the patients had Kappa-type melanoma and 19.1% had Lambda-type myeloma. It was observed that 19,1% of the patients had the IGA type, 51,5% had the IGG type, and 29.4% had a negative immunoglobulin type. According to the R-ISS stage, 2.9% of the patients were stage 1, 61.8% were stage 2, and 35.3% were stage 3 (Table 1).

In our patients, the p53 mutation was observed at the highest frequency among the types of abnormalities, accounting for 45.6%. Additionally, the most common translocation among our patients was identified as t(11;14).

VCD (bortezomib, cyclophosphomide, and dexamethasone) was the most frequently used chemotherapy, provided to 47.1% of patients at our hospital. As a result of the treatments given, 60.3% of our patients did not respond to treatment. In total, 64.7% of our patients with cytogenetic abnormalities died on or before 1 June 2023.

There was no significant difference in treatment response according to gender difference between males and females (*p* = 0.322), kappa–lambda typing (*p* = 0.597), immunoglobulin typing (*p* = 0.072), an age limit of 65 years (*p* = 0,88), or disease stage (*p* = 0.407) (Table 2).

According to the cytogenetic abnormality grouping of our patients according to the t(4;14), t(11;14), del(13q14), and t(11;14) + del(13q14) cytogenetic abnormalities, it was observed that there was no significant difference between our patients with cytogenetic abnormalities in terms of the response to treatment (*p* = 0.322).

In the analysis performed by grouping our patients according to VD, VCD, VRD, and VAD chemotherapies, it was observed that these chemotherapies did not cause a significant difference in terms of response (*p* = 0.729).

When we compared the cytogenetic abnormalities and response rates of our patients, 50% of the patients with a 13q14 deletion responded to treatment.

When the treatment responses of the patients were evaluated through a mean survival analysis, a significant difference was found between the treatment-responsive patients and the non-responsive patients in terms of mean survival (*p* = 0.046).

Differences in the proportions of patients who survived and those who died between gender (*p* = 0.886), age limit of 65 years (*p* = 1.00), Kappa-Lambda (*p* = 1.00), IGA, IGG, and IGneg (*p* = 0.787), and R-ISS stage (*p* = 1.00) and the difference between p53 deletion, t(4;14), t(4;14), t(11;14), del(13q14), and t(11;14) + del(13q14) (*p* = 0.637) were not statistically significant (Table 3).

It was found that the difference between the rates of patients who survived and those who died in response to treatment was not statistically significant (*p* = 0.123).

The median survival time of the patients was independent of the age limit of 65 years (*p* = 0.253), gender (*p* = 0.543), the kappa–lambda ratio (*p* = 0.755), immunoglobulin (*p* = 0.633), cytogenetic abnormalities (*p* = 0.616), chemotherapy (*p* = 0.319), and R-ISS stage (*p* = 0.989).

**Figure 2:**
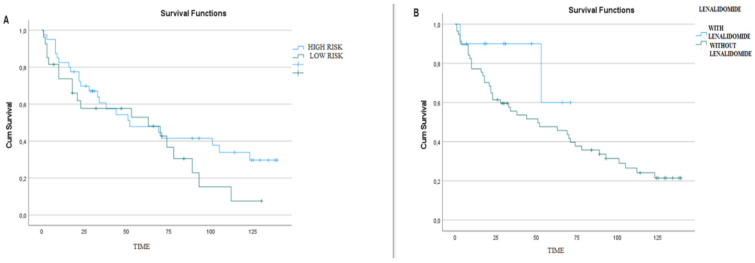
(**A**) Effect of high-risk and low-risk cytogenetic abnormalities on overall survival. (**B**) Lenalidomide-based treatments and their impact on overall survival.

As a result of the analysis of the data obtained, it was observed that the specific cytogenetic abnormalities of the patients and the specific chemotherapies given did not make a significant difference in the treatment response or the mean survival of the patients, so subgroup analyses of response and survival according to cytogenetic abnormality and chemotherapy type subgroups could not be performed. Subgroup analyses could not be performed to determine which treatment option would be more meaningful for which cytogenetic abnormality and which would be effective for average survival. Therefore, cytogenetic abnormalities were classified as high- and low-risk (Figure 2), and chemotherapies were classified as lenalidomide-based, cyclophosphamide-based, and bortezomib-based, and their effects on treatment responses, deceased status, and overall survival were compared.

There were 52 patients receiving bortezomib-based chemotherapy, 16 patients without bortezomib, 32 patients receiving cyclophosphamide-based chemotherapy, 36 patients without cyclophosphamide, 10 patients receiving lenalidomide-based chemotherapy, and 58 patients without lenalidomide.

We had 27 patients who responded to the treatments and 41 patients who did not respond to the treatments.

In the comparison of cytogenetic abnormalities between the high- and low-risk groups (*p* = 0.415), the use of bortezomib-based chemotherapy versus without bortezomib (*p* = 1.00), cyclophosphamide-based chemotherapy versus without cyclophosphamide (*p* = 0.549), and lenalidomide-based chemotherapy versus without lenalidomide (*p* = 0.502) revealed no significant differences in treatment response.

No significant result was found in the comparison of the survival status of our patients between the high-risk, standard-risk, and no-risk groups (*p* = 0.476).

There was no significant result in terms of mortality rates between bortezomib-based treatments and treatments without bortezomib (*p* = 0.199) and between cyclophosphamide-based treatments and treatments without cyclophosphamide (*p* = 0.686) (Table 3).

There was a significant difference in mortality rates between lenalidomide-based treatments initiated at the time of the initial diagnosis and non-lenalidomide-based treatments (*p* = 0.003) (Table 3).

The survival rate was found to be 80% with lenalidomide-based treatments (Figure 1) and 27.58% with non-lenalidomide treatments.

Based on this significant value, lenalidomide-based treatments were analysed in terms of whether they could create a significant difference between high-risk, standard-risk, and no-risk groups in terms of survival.

The difference between the rates of patients with high-risk cytogenetic abnormalities + lenadomide treatment and patients with low-risk cytogenetic abnormalities + lenalidomide treatment was not statistically significant (*p* = 1.00).

In the classification of our patients according to lenalidomide-based chemotherapies, it was observed that starting lenalidomide-based chemotherapies in the initial treatment stage resulted in a significant difference in long-term survival (*p* = 0.026). A 14-fold difference in long-term survival was observed between patients who started treatment with lenalidomide-based chemotherapies and those who received chemotherapy without lenalidomide (Table 4).

## 4. Discussion

The neoplastic proliferation of malignant plasma cells, which results in the formation of heavy- or light-chain monoclonal paraproteins, is the hallmark of MM. MM is a tumour originating from the post-germinal lymphoid B-cell lineage and derived from bone; it is a neoplasm of clonal plasma cells that develops in the marrow after lineage commitment [13,14].

MM is still not a curable disease with current treatment approaches. The pathogenesis of MM is not clear, and choosing a therapy target is an important challenge. Hence, finding more potent targets for MM is necessary [15]. There are genetic subgroups of MM that exhibit variations in clinical outcomes. Life expectancy varies as a result of risk assessments, especially for cytogenetic abnormalities. However, the predictive value of cytogenetic abnormalities is not fully understood and is at the research level [16]. Chromosomal abnormalities in Multiple Myeloma are classified according to risk status [17].

In a study by Chakraborty et al. [18], 31% of the patients were evaluated as having high-risk cytogenetic features. The rate of patients evaluated as having standard-risk cytogenetic features was 63%. In our study, 58.8% of our MM patients had high-risk cytogenic abnormalities and 41.2% had standard-risk cytogenetic abnormalities, which is different from the literature. When the rate of high-risk cytogenetic abnormalities was compared among all cytogenetic abnormalities, t(4;14) was observed in 13.2%. In our study, 58.8% of our MM patients had high-risk and 41.2% had standard-risk cytogenetic abnormalities, which is different from the literature [19]. Variations in disease progression, response to therapy, and patient characteristics among different cytogenetic risk groups may influence survival outcomes.

Among cytogenetic abnormalities, trisomies are the most common. Among translocations, t(11;14) is the most common and is followed by t(4;14) [6]. A variety of secondary chromosomal abnormalities occur as the disease worsens and becomes more proliferative. Secondary aberrations such as del(13q), del(17p), and del(1p) or amplification are frequently observed in these cases [20]. In our study, t(11;14) was the most common translocation, with a rate of 17.6%, and t(4;14) was the second most common translocation, with a rate of 13.2%, which is consistent with the literature.

The majority of chromosome 17 deletions are hemizygous, and in the entire *p* arm, it is a genetic event observed in approximately 10% of new myeloma cases; this frequency increases in later disease stages [21]. In our study, p53 deletion was observed in 45.6% of cytogenetic abnormalities.

According to several findings, patients with trisomies or hyperdiploidy outcomes fared well compared to those with del(17p), del(13q), and t(4;14) [22]. No significant difference was observed between stages in the treatment response comparison of our patients (*p* = 0.407). There was no significant difference between p53 deletion, t(4;14), t(11;14), 13q14 deletion, and t(11;14) + 13q14 deletion (*p* = 0.322). However, when we compared the cytogenetics among themselves, it was observed that the 13q14 deletion mutation responded to the treatments more than the other mutations. We think that the reason why no significant treatment difference was observed between mutations in our patients was due to the small number of patients and the fact that the groups were not homogeneous.

There was no significant difference in response to treatment between VD, VCD, VRD, and VAD (*p* = 0.729). A significant difference was observed in the OS comparison of our treatment-responsive patients (*p* = 0.046). The mean survival of our patients who responded to the treatments given was 78.5 months, while the mean survival of our patients who did not respond to the treatments was 51.6 months.

There was no significant difference in OS when the median survival of our patients was classified according to VD, VCD, VRD, and VAD chemotherapy groups (*p* = 0.319). When we compared the chemotherapies among themselves, the OS of the patients treated with VAD chemotherapy was 22.8 months, and the mean survival of the patients treated with VAD chemotherapy was the lowest. The fact that OS or other comparisons are not meaningful in relation to treatment is due to the reimbursement policies in Turkey and the difference in treatment algorithms. Prospective study analyses are needed for an ideal analysis.

There was no significant difference in the response rates of our patients in the high-standard and no-risk groups (*p* = 0.415). In a study conducted by Avet-Loiseau et al. in 2010 [23], partial improvement in the t(4;14) mutation was reported in patients receiving only bortrzomib.

In our study, no significant difference was found between the bortezomib and without bortezomib chemotherapy groups in terms of response rate (*p* = 1.00).

There was a significant difference in patient mortality rates between lenalidomide-based treatments and treatments without lenalidomide initiated during the initial stage of diagnosis (*p* = 0.003). The mortality rate of patients who started lenalidomide-based treatments was 20%, while the mortality rate of patients who started without lenalidomide was 72.4%.

A comparative logistic progression analysis of all values of our patients showed that lenalidomide-based treatments were significantly different from lenalidomide-free treatments at the beginning of treatment and resulted in 14-fold longer survival (*p* = 0.026). Phase 3 randomised controlled trials in some clinics have shown that lenalidomide maintenance therapy has a positive effect on progression-free survival and overall survival [24,25].

In a study conducted by Rosiñol et al., it was observed that the use of proteasome inhibitors provoked a more significant response to treatment in patients with high-risk cytogenetic abnormalities with t(4;14) [26]. Another clinical trial and meta-analysis confirmed that bortezomib-based induction provides better results compared to non-bortezomib-based induction but does not fully overcome the unfavourable prognostic impact of cytogenetic abnormalities [27,28]. Clinicians in China favour maintenance therapies such as lenalidomide or bortezomib in high-risk situations and have shown that these therapies are more effective than thalidomide in non-transplant myeloma patients. High-risk cytogenetic abnormalities were observed to be more frequent in patients on lenalidomide or bortezomib, and extended maintenance treatment regimens improved survival [29]. In a study conducted by Puertas et al. [30], MM patients with t(11;14) did not benefit as much from the introduction of new agents as patients with standard risk, suggesting that other treatments are needed to improve outcomes.

We conducted our study on patients with cytogenetic abnormalities. Data on 68 of 98 patients with cytogenetic abnormalities were obtained due to the difficulty of keeping a regular archive at our hospital and inadequacies in follow-up and treatment. In our study, it was found that chemotherapy differences in the treatment response of cytogenetic abnormalities were not significant, and sub-analyses were not performed to determine which cytogenetic abnormality required specific chemotherapy. We think that this is due to the fact that the data of 68 out of 98 patients with stiogenetic abnormalities in our hospital were obtained and the number of chemotherapy and cytogenetic type groups to be analysed could not be reached. The results of the analyses show that lenalidomide-based treatments had a 14-fold longer effect on median survival in patients with cytogenetic abnormalities.

Possible reasons for the low survival rates could include disease aggressiveness, the presence of cytogenetic abnormalities, delayed diagnosis, comorbidities, and variations in treatment responses among patients. 

There were limitations to our study; being retrospective in nature, research is susceptible to inherent biases and confounding factors that may influence the results. The lack of randomization and heterogeneity within the study groups could affect the validity and generalizability of our findings. Furthermore, the relatively small sample size may limit the statistical power and precision of our analyses, leading to potential underestimations or overestimations of treatment effects. Additionally, our study focused on a single type of population which may not fully represent the diversity of patients with Multiple Myeloma. Despite these limitations, our study provides valuable insights into the efficacy of lenalidomide-based treatments in patients with cytogenetic abnormalities in multiple myeloma. Further research with larger, prospective, and more diverse cohorts is warranted to validate our findings and elucidate optimal treatment strategies for this patient population.

## 5. Conclusions

Our study is an evaluation of the cytogenetic characteristics of Multiple Myeloma patients. Our findings show that high-risk cytogenetic abnormalities were generally absent among our patients, and there were no significant differences in treatment response. However, we observed that patients with a 13q14 deletion responded to treatment at a higher rate, and lenalidomide-based therapies improved overall survival.

## Figures and Tables

**Table 1:** The demographic and clinical characteristics, cytogenetic abnormalities, chemotherapies, responses, and survival fractions of patients.

Age Median (Min–Max)		59.0 (29–84)	
		*n*	%
Sex	Female Male	25 43	36.8 63.2
Age groups	<65 years ≥65 years	22 46	32.4 67.6
Light-chain status	Kappa Lambda	55 13	80.9 19.1
Ig type	Ig A Ig G Negative	13 35 20	19.1 51.5 29.4
ISS stage	Stage 1 Stage 2 Stage 3	2 42 24	2.9 61.8 35.3
Cytogenetic Abnormalities	t(4;14) t(11;14) del(13q14) t(11;14) + del(13q14)	9 12 12 4	13.2 17.6 17.6 5.9
Chemotherapies	VD VCD VRD VAD	10 32 10 16	14.7 47.1 14.7 23.5
Responses	Response No response	41 27	60.3 39.7
Survival Function	Alive Deceased	24 44	35.3 64.7

Ig, immunoglobulin; ISS, International Staging System; VD, bortezomib–dexamethasone; VCD, bortezomib–cyclophosphamide–dexamethasone; VRD, bortezomib–lenalidomide–dexamethasone; VAD, vinkristin–doxorubicin–dexamethasone.

**Table 2:** Assessment of treatment responses.

		Treatment Response n (%)		*p*
		Present n: 27	Absent n: 41	
Gender	Female Male	8(29.6) 19(70.4)	17(41.5) 24(58.5)	0.322
Light-Chain Status	Kappa Lambda	21(77.8) 6(22.2)	34(82.9) 7(17.1)	0.597
IG Type	IgA IgG Negative	5(18.5) 18(66.7) 4(14.8)	8(19.5) 17(41.5) 16(39.0)	0.072
Age Subgroup at Diagnosis	<65 years ≥65 years	18(66.7) 9(33.3)	28(68.3) 13(31.7)	0.88
ISS Stage	Stage 1 Stage 2 Stage 3	1(3.7) 19(70.4) 7(25.9)	1(2.4) 23(56.1) 17(41.5)	0.407 *
Genetic Abnormalities	p53 t(4;14) t(11;14) del(13q14) t(11;14) + del(13q14)	15(55.6) 3(11.1) 2(7.4) 6(22.2) 1(3.7)	16(39.0) 6(14.6) 10(24.4) 6(14.6) 3(7.3)	0.322 *
Treatment Initiated	VD VCD VRD VAD	5(18.5) 11(40.7) 5(18.5) 6(22.2)	5(12.2) 21(51.2) 5(12.2) 10(24.4)	0.729 *
Follow-up Period		78.5 months	51.6 months	**0.046**

Ig, immunoglobulin; ISS, International Staging System; VD, bortezomib–dexamethasone; VCD, bortezomib–cyclophosphamide–dexamethasone; VRD, bortezomib–lenalidomide–dexamethasone; VAD, vinkristin–doxurobusin–dexamethasone. *: Fisher’s exact test, x: independent-samples *t*-test.

**Table 3:** Mortality rates of patients according to different features.

		Mortality n (%)		
		No n: 24	Yes n: 44	*p*
Age groups	<65 years ≥65 years	7(29.2) 17(70.8)	15(34.1) 29(65.9)	0.886
Sex	Female Male	9(37.5) 15(62.5)	16(36.4) 28(63.6)	1.000
Light-Chain Status	Kappa Lambda	19(79.2) 5(21.8)	36(81.8) 8(18.2)	1.000
IG Type	Ig A Ig G Negative	5(20.8) 11(45.8) 8(33.3)	8(18.2) 24(54.5) 12(27.3)	0.787
ISS Stage	Stage 1 Stage 2 Stage 3	1(4.2) 15(62.5) 8(33.3)	1(2.3) 27(61.4) 16(36.4)	1.000
Genetic Abnormalities	P53 t(4;14) t(11;14) 13q14 del t(11;14) + del(13q14)	12(50.0) 4(16.7) 5(20.8) 2(8.3) 1(4.2)	19(43.2) 5(11.4) 7(15.9) 10(22.7) 3(6.8)	0.637
Treatment	VD VCD VRD VAD	3(12.5) 10(41.7) 8(33.3) 24(100.0)	7(15.9) 22(50.0) 2(4.6) 44(100.0)	0.014
Treatment Response	Response No response	13(54.2) 11(45.8)	14(31.8) 30(68.2)	0.123
Cytogenetic abnormality risk	High risk Low risk	16(66.7) 8(33.3)	24(54.5) 20(45.5)	0.476
Survival rate according to treatment	With bortezomib Without bortezomib	21(87.5) 3(12.5)	31(70.5) 13(29.5)	0.199
	With cylophosphamide Without cylophosphamide	10(41.7) 14(58.3)	22(50.0) 22(50.0)	0.686
	With lenalidomide Without lenalidomide	8(33.3) 16(66.7)	2(4.5) 42(95.5)	**0.003**

Ig, immunoglobulin; ISS, International Staging System; VD, bortezomib–dexamethasone; VCD, bortezomib–cyclophosphamide–dexamethasone; VRD, bortezomib–lenalidomide–dexamethasone; VAD, vinkristin–doxurobusin–dexamethasone. Chi-squared/Fisher’s exact test was applied. *p* < 0.05 is considered statistically significant.

**Table 4:** Lenalidomide-based treatments and their impact on overall survival.

	Mean Survival Time				*p*-Value
	Estimate	S.D.	%95 CI		
			Lower Bound	Upper Bound	
Lenalidomide (+)	58.800	7.351	44.392	73.208	0.197
Lenalidomide (−)	63.262	6.978	49.586	76.939	

A Kaplan–Meier survival analysis was conducted, and *p* < 0,05 was considered statistically significant. CI: confidence interval.

## Data Availability

All relevant data are within this manuscript.
